# Complete genome sequences of *Lacticaseibacillus paracasei* strains E and E11.5 isolated from dairy products

**DOI:** 10.1128/mra.00120-25

**Published:** 2025-06-12

**Authors:** Nina Zhang, Guesh Mulaw, Antti Karkman, Timo M. Takala, Per E. J. Saris

**Affiliations:** 1 Department of Microbiology, Faculty of Agriculture and Forestry, University of Helsinki, Helsinki, Finland; 2 Biology Department, College of Natural and Computational Sciences, Aksum University, Aksum, Ethiopia; University of Maryland School of Medicine, Baltimore, Maryland, USA

**Keywords:** DNA sequencing, *Lacticaseibacillus paracasei*, *de novo *assembly

## Abstract

*Lacticaseibacillus paracasei* is a lactic acid bacterium commonly present in various dairy products and known to have probiotic properties. Here, we report the complete genome sequences of two *L. paracasei* strains isolated from dairy products in Finland and Ethiopia. Both strains contain about 3 Mb chromosomes with several plasmids.

## ANNOUNCEMENT

*Lacticaseibacillus paracasei* is a lactic acid bacterium commonly found in a range of fermented milk products (1). Here, we report the whole-genome sequences of two *L. paracasei* strains. The strain E was isolated in 1964 from cheese made in an industrial dairy of Valio Ltd, Finland, and later, we studied the strain as a food-grade gene expression host for cheesemaking (2, 3). The strain E11.5 was isolated in 2017 using the method described by Goa et al. (4), from ergo, a yogurt-like product made by spontaneous fermentation in Ethiopia.

Strains were cultured overnight in MRS broth at 37°C. The total DNA of the strains was obtained using the MagAttract HMW DNA Kit (Qiagen, Hilden, Germany). DNA concentration was measured by NanoDrop One (Thermo Scientific, Waltham, MA, USA). DNA was sheared using the Bullet Blender Storm Pro (Next Advance, Inc., USA). The library was constructed by using the SMRTbell Prep Kit 3.0 (Pacific Biosciences, Menlo Park, CA, USA). After additional AMPure bead purifications and DNA damage repair treatment (Pacific Biosciences, Menlo Park, CA, USA), the completed library was sequenced on the PacBio Sequel II. Nucleic acid integrity was assessed by the Agilent Fragment Analyzer system (Agilent, Santa Clara, CA, USA). SMRT Link v11.1, machine-built-in software, was used to demultiplex barcodes, trim adapters, and generate HIFI reads. The quality of sequence data was evaluated with FastQC version 0.11.9 (5). Default parameters were used except where otherwise noted. The genomes were assembled by Flye version 2.9.2 with PacBio-HiFi option (6) and Unicycler version 0.5.0 (7). Unicycler was used for assembling smaller plasmids that Flye might have missed (8). Bandage version 0.8.1 (9) was used to evaluate the assembly graphs. The alignment between the original sequence reads and the assembly was performed using minimap2 version 2.28 (r1209) (10), sorted by SAMtools version 1.21 (11), and subsequently visualized with IGV version 2.16.2 (12).

The genomic assembly quality was evaluated by QUAST version 5.2.0 (13). Genome completeness was evaluated by CheckM2 version 1.0.1 (14). Assemblies were annotated with the NCBI automatic Prokaryotic Genome Annotation Pipeline (15). The taxonomy of the strains was assigned by GTDB-Tk version 2.3.2 with reference data version r214 (16).

For strain E, Flye generated five circular non-chromosome contigs, whereas Unicycler assembled nine non-chromosome contigs, of which six were circular and three linear. Upon aligning the contigs to themselves by NCBI BLASTN (17), it was observed that linear contigs 2 and 3 contained flanking repetitive regions. When these repeated regions were removed, the contigs were identical to those assembled by Flye. We adopted the Unicycler-assembled contigs 4, 5, 7, 8, 9, and 10 and removed contig 6 after carefully examining read quality and distribution for the final version of the genome. For the strain E11.5, both assemblers gave five contigs. However, there was a discrepancy on one non-chromosome contig 5. The bigger contig generated by Flye displayed flanking repeat regions. Similarly, we scrutinized the sequence reads and chose the Unicycler assembly. The reads and assembly data are shown in Table 1.

**TABLE 1:** Summary data for the sequenced strains

Strain	Long-read sequencing technology	Number of CCS reads	Reads *N*_50_ (bp)	Coverage (×)	Number of contigs	Assembly chromosome size (bp)	Assembly *N*_50_ (bp)	Plasmid sizes (bp)	GC content (%)	CheckM completeness (%)	GTDB species assignment	Number of CDSs	Number of rRNAs	SRA accession no.	GenBank accession no.
E	PacBio HiFi	45,876	14,645	147	9	2,978,984	2,978,984	78,009 60,563 40,929 37,754 9,346 6,997 5,345 4,307	46.1	99.86	*Lacticaseibacillus paracasei* subsp*. paracasei*	3,065	15	SRS22907183	CP171623– CP171631
E11.5	PacBio HiFi	837,376 subset (100,000)	11,068	584	5	3,063,221	3,063,221	44,793 40,816 30,201 11,830	46.4	99.97	*Lacticaseibacillus paracasei*	2,972	15	SRS23039223	CP172438 –CP172442
